# Roadblocks for integration of novel biomarker concepts into clinical routine: the peptoid approach

**DOI:** 10.1186/alzrt253

**Published:** 2014-04-30

**Authors:** Hugo Vanderstichele, Thomas Kodadek

**Affiliations:** 1 ADx Neurosciences, Technologiepark 4, 9052 Gent, Belgium; 2 Department of Chemistry, The Scripps Research Institute, Scripps Florida, 130 Scripps Way, Jupiter, FL 33458, USA; 3 Department of Cancer Biology, The Scripps Research Institute, Scripps Florida, 130 Scripps Way, Jupiter, FL 33458, USA

## Abstract

In the field of Alzheimer’s disease, the development of novel biomarker assays is critically needed to improve the early diagnosis of the disease, to estimate the risk of developing the disease, to predict the rate of cognitive decline, and to monitor the response or effectiveness of a therapy. The molecular mechanisms of the disease are becoming more evident. This basic knowledge has yet to be translated into novel biomarker tools with a clinical value for general use by the community. There is therefore high interest in evaluating new technological approaches beside the classical immunoassay approach. The present paper discusses the hypothesis that there might be an adaptive immune response, unique to Alzheimer’s disease, which can be visualized by the presence in body fluids of antibodies against specific analytes. Current technologies to identify such antibodies are reviewed. In addition, the major challenges to transfer discovery results of the novel antibody-based biomarker assays to a clinically relevant test will be discussed.

## Introduction

Alzheimer’s disease (AD) is the most common type of dementia. AD is a heterogeneous and multifactorial disease that is characterized by a progressive cognitive decline. The main risk factor for AD is age, affecting 11% of people over the age of 65 years and 32% of people 85 years of age or older. The already tremendous cost of this disease in developed countries will only increase as the population ages [1].

The pharmaceutical industry faces a challenge to develop disease-modifying drugs that are able to stop or slow down AD at a very early phase. One of the greatest barriers is the identification of appropriate patients to include in clinical trials. It is now well accepted that pathological processes (for example, formation of neurofibrillary plaques and tangles, synapse loss, inflammation, oxidative stress) are operative in the brains of AD patients years and even decades prior to the development of symptoms [2,3]. By the time overt symptoms arise, it is probably too late for many classes of drugs to have a clear therapeutic benefit even if they slow down the neurodegenerative disease process.

Today, quantification of changes in the concentrations of tau and amyloid-beta (Aβ) proteins in cerebrospinal fluid (CSF) has the highest clinical value for dementia diagnosis. These markers reflect ongoing pathology (plaques, tangles) in the brain and identify persons at risk of developing the disease. Unfortunately, although there is a sequential change of biomarker signature over time [2,4], the CSF markers have only limited use as progression markers. In addition, worldwide integration of this first generation of CSF biomarker immunoassays into clinical routine testing is hampered by analytical issues (for example, inter-center variability, dilutional linearity, absence of reference methods) [5-7]. No US Food and Drug Administration-approved assays for AD biomarkers are currently available. Nonetheless, using these methods in combination with various phenotypic examinations, experienced centers in the field of AD can deliver a diagnosis of AD with a clinical sensitivity and specificity of 85% for subjects with dementia [1], but the results can be much poorer in a typical clinical setting. In addition, there is no direct link available between the levels or changes in the levels of these biomarkers and the cognitive state or daily living activity of a patient.

Clearly, the AD field urgently needs new biomarkers and reliable, clinically viable biomarker assays to measure them. In other words, the biomarker must be obtained using a relatively non-invasive sampling technique, such as a blood draw, and quantified reproducibly in many clinical centers. The assay to monitor the biomarker level must have good precision, no matrix interference problems, limited workload, and provide a concentration that is linked to values obtained using an internationally accepted reference method. An ideal biomarker assay would allow early detection of the disease, its consequences, and also provide a differentiation between AD and several other types of dementia that can exhibit similar symptoms but occur via different mechanisms. Finally, this assay, when repeated over time, would need to provide information on the progression rate of the disease or the rate of cognitive decline. It seems unlikely that a single biomarker would be sufficient to fulfill all of these needs. Therefore, one assumes that the ultimate assay type will monitor the levels of several biomarkers simultaneously.

**Figure 1:**
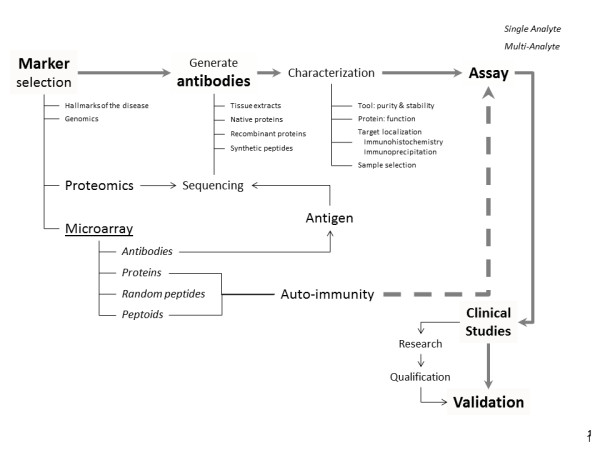
Schematic presentation of different approaches to develop novel biomarker assays.

Unfortunately, despite tremendous investment, over the last 15 years no new biomarkers have been qualified to the same extent as the CSF tau and Aβ proteins. The search for blood-based biomarkers with a direct link towards the pathology in the brain using the classical immunoassay approach (depicted in Figure 1) was even more difficult than using CSF and required complex protein signatures [8]. The proportion of brain-specific proteins in blood is much lower than that in the CSF. Transfer of proteins from brain to blood might be very low or related to blood–brain barrier deficits. Most of the proteins identified as biomarkers for use in the field of AD are also measurable in samples obtained from healthy subjects. One must therefore be able to quantify, with high precision and accuracy, a small change in the concentration or conformation of the analyte in a complex mixture of proteins. The presence of both systematic error (bias) and random error in the detection methodology by confounding factors not necessarily known to the field can significantly influence the output [9]. Clearly, the problem for the classical immunoassay approach remains challenging.

In this review, we consider a novel potential source of AD-specific biomarkers: the adaptive immune system. Progress from several laboratories attempting to identify antibodies linked to AD is reviewed. We also discuss some of the technological challenges to transition the discovery results into diagnostic assays that could be integrated into routine clinical laboratory testing. The studies described in the present review need to be considered as feasibility studies, aiming at the design of prototype assay formats (including, but not limited to, the selection of critical raw materials and sample type, test procedures, and showing proof of concept at the clinical level). It is important to mention that the pitfalls described in this review paper are not unique to any particular method or marker, or even to AD, but to immunoassay development in general.

## Biomarker assay development: the classical approach

The CSF proteins currently accepted as useful markers for AD diagnosis have been isolated from the hallmark brain lesions that were observed at autopsy. Extracts from these lesions were used to immunize animals to generate antibodies with high affinity for proteins present in these preparations, potentially allowing for the development of an immunoassay capable of measuring low levels of the antigens (Figure 1). Following isolation of the newly generated antibodies, the targets were identified by mass spectrometry or other molecular technologies after immunoprecipitation. In so doing, researchers might have inadvertently overlooked other proteins that are directly pathogenic (that is, contribute directly to the formation of neuropathology) or that are not detected in great quantities in the tissues on postmortem examination. As such, the classical approach documents the clinical value of a specific assumption after generation of antibodies, followed by development of immunoassays.

The time needed to translate a biomarker concept into a product with proven clinical value can be short (several months) or longer (more than 2 years). The development time includes the clinical verification of several versions of the product and is in part related to the need to use retrospectively or prospectively collected samples from well-diagnosed subjects to show the clinical utility of the new biomarker or biomarker assay. Standardization of assays at the pre-analytical, analytical, or post-analytical level can be complex and labor intensive [10]. Extensive characterization by functional and nonfunctional assay formats of the analytes that are captured by the antibodies in the assay format is essential and needs to be done in an early phase of the development process in order to obtain a correct interpretation of the outcome of the test. Some of the interferences in the assay formats might be derived from auto-antibodies or protein interferences at the site of the epitope of the antibodies included in the sandwich immunoassay.

## Blood-based biomarkers: the search for Alzheimer's disease-specific antibodies

Autoimmunity against self-proteins is a new emerging field of biomarker research in AD. The adaptive immune system responds to the presence of foreign antigens of various infectious agents, in part, by producing large amounts of antibodies that bind to them. Many studies revealed that the definition of foreign antigens is much broader than molecules from bacterial or viral pathogens [11]. The most optimistic view along these lines would suggest that the immune system recognizes continuously any disease state that produces unnatural molecules, such as abnormally modified proteins, which are not present under normal physiological conditions. If so, these adaptive immune responses could represent a starting point for generation of useful biomarkers [12]. Moreover, from an analytical point of view, the fact that the adaptive immune response produces many antibodies for each molecule of antigen makes antibodies a potentially easier biomarker to detect than the antigen it reacts against.

There have been reports in the literature of the presence in patients of antibodies against molecules, such as Aβ42, thought to be involved in the pathophysiology of AD [13,14]. These antibodies could be protective, ameliorate the cognitive decline, and become enriched in cognitively normal older people, but not in AD patients. This view is supported by data from ongoing clinical trials with Aβ-clearing antibodies [15,16]. Technical problems with their detection (for example, lack of detailed information on the candidate antigens, low concentrations in biological fluids, low affinity for the analyte of interest, presence of antigen–antibody complexes together with free analyte) hampered the translation of the concept into a diagnostic assay with clinical potential [17,18]. Advances in proteomics technologies have opened the door to conducting more unbiased research towards these putative biomarkers.

## The search for novel Alzheimer's disease biomarkers using protein microarray technology

Advances in high-throughput cloning and protein expression have made it possible to display thousands of different peptides or (recombinant) proteins in an array format [19]. Some of these arrays were integrated in exploratory studies to identify autoreactive antibodies in blood samples of patients with autoimmune diseases [20].

Nagele and coworkers employed a commercially available proteome array, displaying several thousands of recombinant human proteins, to search for the presence of autoantibodies with a link to AD [21] or Parkinson’s disease [22]. Screening was accomplished by detection of reactivity towards the displayed sequences on the array in serum samples from either nondemented controls or AD patients. The method includes a wash step to remove nonspecifically bound antibodies, after which the binding of serum IgG antibodies was visualized with a fluorescent-labeled anti-human IgG secondary antibody. The authors reported that 451 proteins on the array retained significantly more antibody responses from AD sera than control sera. Interesting to note is that autoantibodies could even be visualized in the healthy control population. The 10 most promising protein–IgG complexes were selected for a follow-up qualification study using serum samples from 50 AD patients and 40 healthy subjects. Using an algorithm to weight the contributions of the 10 markers, the 10-plex panel test was able to discriminate AD from control samples with a sensitivity and a specificity of 96% and 92%, respectively. In an additional study, the authors reported a separation between AD patient-derived samples and those collected from Parkinson’s disease patients, especially using pentatricopeptide repeat domain-2 protein and FERM domain-containing 8. However, although there was a statistically significant difference in the immunoreaction to these two proteins between serum of AD and control individuals, it would be very difficult to employ the markers individually for definitive diagnosis.

Interestingly, none of the 10 best discriminator proteins identified in Nagele and colleagues’ study have any known role in AD. For example, no antibodies against Aβ, known to be present in blood samples, were identified. The latter observation could perhaps be explained by the fact that the control subjects in the study design already had preclinical AD, including the presence of anti-Aβ antibodies. The authors hypothesized that the AD-related autoimmune reaction is the result of a chronic neuronal death, exposing intracellular proteins to the immune system, where they are recognized as antigenic. This idea explains why there are so many autoantibodies that are (to some extent) enriched in AD patients and why the markers are not immediately linked mechanistically to the disease process. Presently unclear is how one could differentiate AD from other neurological disorders if the result to the abovementioned explanation is correct. Perhaps different cell populations are killed in different neurodegenerative diseases, resulting in a somewhat different autoantibody profile. One can speculate also that antibodies exist in nondemented older people that help them to resist the disease by clearance of toxic extracellular molecules such as Aβ oligomers, but are present in such low concentrations in a sample that more sophisticated technologies or sample preparation procedures are required for their measurement. Nevertheless, this promising study is consistent with the idea that the immune system detects AD, and supports the initiation of much larger blinded validation trials of the 10 marker set.

## The search for an Alzheimer's disease biomarker biosignature using random peptide arrays

Another array-based approach to the discovery of useful IgG markers to AD was reported by Restrepo and colleagues [23]. In this case, an array of approximately 10,000 randomly selected peptides was employed rather than recombinant proteins. The team generated an array of 20 random residue sequences, the vast majority of which would not be found in the human proteome. The goal of the study was not to identify potential native peptide epitopes that are recognized by the AD autoantibodies, but to use the peptide sequences as a random collection of potential ligands to determine whether the fingerprint or immunosignature of total IgG antibody binding to the array would be of any diagnostic utility for AD. The authors found that plasma samples collected from AD patients had a common fingerprint, different from fingerprints in samples derived from cognitively normal patients or from one person with progressive supranuclear palsy. A blinded follow-up analysis of eight random samples, using the patterns gleaned from the open-label samples, mostly correctly identified the samples. The identities of most of the antibodies that produce the signature are not known. A blocking experiment that prevented anti-Aβ40 antibodies from binding to the array indicated that only a small portion of the immunosignature was due to these antibodies.

## The search for novel Alzheimer's disease biomarkers using synthetic antigen surrogates

**Figure 2:**
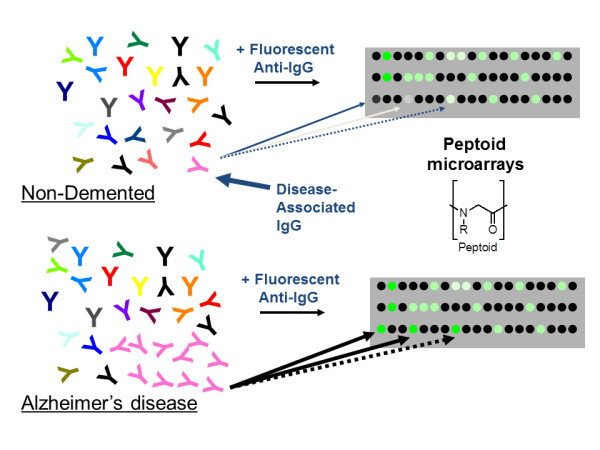
**Schematic presentation of the process employed to identify peptoids that bind to antibodies present at higher levels in Alzheimer’s disease patients.** Serum samples were hybridized to an array of thousands of eight-mer peptoids spotted covalently onto chemically modified glass microscope slides. After washing and addition of a fluorescent-labeled secondary antibody, the signals were read at each spot. Peptoid spots that consistently captured at least three times more IgG antibodies from the serum of the Alzheimer’s disease patients than that from controls were sequenced by mass spectrometry to identify the structure.

A third array-based approach to AD autoantibody discovery was reported by Reddy and colleagues [24]. An array of thousands of unnatural molecules, called peptoids [25] (oligomers of N-substituted glycines), was produced (Figure 2). Since the peptoids are completely unnatural molecules, they cannot resemble a native antigen to any significant extent. The slides for the peptoid approach were created via a stepwise production process. The most critical part is the coating of the glass surface with a thick layer of polyethylene glycol oligomer to reduce nonspecific antibody binding to the surface, which is terminated by a maleimide group. The peptoid is then attached covalently to the surface by spotting a small amount of the compound with a carboxy-terminal cysteine onto the array using the same pin-based spotting robots that were employed to create the earliest DNA microarrays [26].

This peptoid technology aims to target antigen-binding sites with a large and chemically diverse set of unnatural molecules in analogy to the development of drugs that target the substrate-binding pockets (active site) of enzymes, but do not strictly mimic the substrate. In contrast to classical immunoassays, but in common with the peptide array work of Restrepo and colleagues [23], this method to search for antigen surrogates does not require prior knowledge of the native antigen. The study by Reddy and colleagues aimed to identify markers that individually discriminated cases from control samples in a small discovery set (AD, *n* = 6; nondemented, aged-matched controls, *n* = 6; Parkinson’s disease, *n* = 6) [24]. Three peptoids were identified from a collection of 4,608 molecules that captured at least five times more IgG antibodies from all six of the AD samples than any of the control samples. Depletion experiments revealed that two of the three peptoids bind the same antibodies, while the third binds a distinct antibody. In a small qualification set of approximately 50 patients, these peptoids individually provided excellent diagnostic sensitivity and specificity (higher than 90%) to identify subjects with AD, even when evaluated as single proteins.

These potential biomarkers are in the process of being validated in larger sample sets. It is not yet clear whether these additional results will justify the translation of this observation into biomarker assays for integration into further clinical development.

## The search for ligands to known proteins using the peptoid approach

Besides the search for autoantibodies, the peptoid technology was used more recently by Luo and colleagues to discover ligands to a known hallmark of AD, Aβ42 [27]. They synthesized 38,416 unique peptoids on beads. The generated peptoid library was screened and several Aβ_42_-selective peptoid ligands were identified. Inhibitor of amyloid (IAM)1 and its dimeric form (IAM1)_2_ were validated as specific Aβ_42_ ligands with anti-aggregation and neuroprotective properties. IAM1 and the dimeric form (IAM1)_2_ were synthesized and evaluated in a variety of biochemical assays. (IAM1)_2_ had a higher affinity for Aβ_42_ than IAM1. IAM1 and (IAM1)_2_ were able to inhibit the aggregation of Aβ_42_ in a concentration-dependent manner. (IAM1)_2_ protected primary hippocampal neurons from Aβ-induced toxicity *in vitro*. Both ligands hold promise as Aβ_42_ detection agents and lead compounds for the development of AD therapeutic agents.

The screening for binding proteins against Aβ (or tau proteins) with the peptoid method could be of help in the search to standardize biomarker testing in CSF and blood. Removal of the binding protein or a block in the interaction with the key proteins could result in more accurate values determined in patient samples, eliminating possible confounding factors for the biomarker testing.

## The transfer of the biomarkers from the laboratory to the clinic

All of the studies mentioned above provide preliminary, but encouraging, results with respect to the future development of antibody-based biomarker assays for AD. Larger qualification studies, followed by validation studies, will extend the information on their true diagnostic value in the clinic.

A distinction must be made between a biomarker and a biomarker assay. Biomarkers have to be qualified and properly validated before they can be used in the clinic routinely. But the applicability of qualified biomarker assays relies on the development of robust assay formats, which is a continuous improvement process, to be initiated in the discovery phase of the project for a specific intended use. Otherwise, there is a risk in a late(r) phase of the development project that clinical utility cannot be confirmed when the assays are transferred from one technology to another technology or when there is a need for scale-up of the production process. Standardization of the assay format for biosignatures (multiplex mode) may be challenging, since optimization of the analytical and clinical performance of one analyte can have a detrimental effect on the performance of another analyte. However, since all of the analytes in this approach are antibodies, this issue may be less problematic than is usually the case for multiprotein tests.

With respect to AD, one can imagine that this development process will occur in at least two phases. The first phase will include the development of an accurate and reliable test for prodromal AD, not necessarily capable of being applied to a large number of samples. This phase will guide the selection of the most appropriate pre-symptomatic or very early AD patients for clinical trials. If a drug becomes available, there will be a critical need in a second phase for a highly effective, accurate, and inexpensive assay that can be employed as a screening test for many millions of people in their middle-aged years (high-throughput screening).

### Comments linked to the use of peptoids

Peptoids have the advantage of their ease of library synthesis via solid-phase split and pool chemistry. The submonomer synthetic route allows the incorporation of unnatural side chains that are different from those of the proteogenic amino acids (different from peptide libraries). The array-based process allows a comparison of results from individual case and control samples, using screening of large libraries, although peptoids are unlikely to form high-affinity interactions with the antibodies. The screening is limited to a few thousand peptoids and requires high-tech microarray technology. The throughput is low. High precision (low variability in the outcome) is often difficult to obtain, hampering the confirmation of the obtained data. Method development often employs a pool of samples, vulnerable to results being dominated by a highly abundant antibody present in only one or a few patient samples in the pooled sample.

### Comments linked to antibody-screening assays

**Figure 3:**
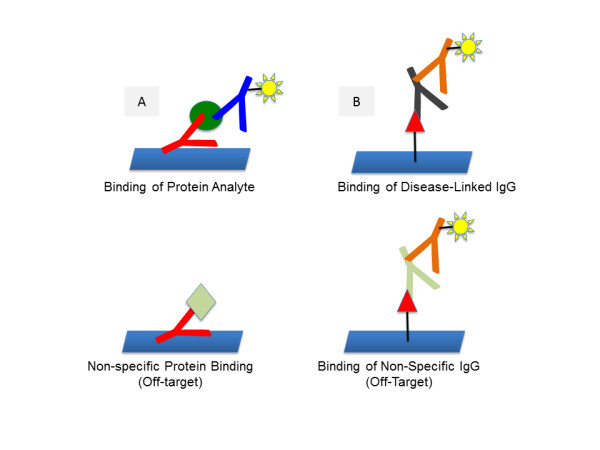
**Schematic representation of classical immunoassays that measure levels of nonantibody serum proteins and an assay that measures levels of antibody analytes.** Schematic representation of **(A)** classical immunoassays that use antibody reagents to measure the levels of nonantibody serum proteins and **(B)** an assay that measures levels of antibody analytes. In (A), off-target binding of a nondisease-related protein will usually not generate a signal since the sandwich antibody is unlikely to bind to this protein. However, any nonspecific binding of an IgG ligand to nondisease-related antibodies will generate a signal, since the binding of any IgG is registered by the labeled secondary antibody. This places a high premium on selective ligands.

The development of assays based on serum antibodies themselves as the analyte may be much easier to make than classical immunoassays. While matrix interference will still be a problem, there is no need for matching several combinations of antibodies. The protein, peptide, or peptoid ligand is immobilized on the solid phase and binds to the antigen-binding site of the antibody, and a labeled secondary antibody then binds elsewhere on the IgG surface to provide the signal. On the other hand, a potential drawback will be error due to off-target binding of nondisease-associated antibodies to the immobilized probe (Figure 3). In a sandwich assay format, one can rely on binding selectivity of two different antibodies, greatly reducing false signals if the antibodies are highly optimized. However, the binding selectivity of small molecules to proteins, including peptides and peptoids, may be nonoptimal and the signal detected will reflect both specific and nonspecific IgG binding events.

### Comments linked to the production process

One can question whether it is practical to use the same complex analytical platform in the discovery phase as well as for the large-scale validation studies. One should thus always bear in mind that even the most promising discovery studies face a challenge in adapting the antibody capture agents (proteins, peptides or peptoids) to more practical analytical platforms.

For example, the utility of ADP3, one of the peptoids that showed promising results on microarrays, was evaluated after immobilization on commercially available maleimide-activated ELISA plates. The ability of this assay format to distinguish between case and control subjects was disappointing and inferior to data obtained using coating of the peptoids on glass arrays. This was due to an unacceptably high level of nonspecific binding to the peptoid–plastic surface. Competition experiments with an excess of soluble ADP3, aiming to block the antigen binding site at the serum antibodies prior to introducing the sample onto the ELISA plate, showed only a small reduction in signal (Busby S, Busby J, Dean S, Kodadek T, 2013, unpublished observation), indicating that most of the signal was not due to specific and reversible ADP3–IgG binding.

Additional experiments suggested that the difference between the utility of ADP3 on glass slides and ELISA plates is related to the fact that ADP3 is a weak ligand for the antigen-binding site of the AD-specific antibodies (Busby S, Busby J, Dean S, Kodadek T, 2013, unpublished observation) and requires the very low background of the polyethylene glycol-modified glass surface to display an acceptable signal-to-noise ratio. This was not completely unexpected, since ADP3 is a primary screening hit from a random collection of a few thousands of peptoids. Unfortunately, it is unlikely that the glass slide array-based display of peptoids used in the discovery study could be translated to a high-throughput clinical assay due to the complexity and modest reproducibility of slide preparation and mechanical compound spotting.

Other possibilities to improve the assay method performance could have been considered, such as a capture-type ELISA, bead-based multiplex technologies, multiplexed line immunoassays or barcodes. However, it was not the aim of the present review paper to explore all the pros and cons of each technology platform individually. The general point is that care must be taken not to overestimate the value for an observation obtained in a research setting, using a small number of samples. Prior to the initiation of scale-up of the production process, a detailed description of the analytical performance of the assay is required, so that all different sources of measurement bias and variability are known and can guarantee the use of the assay as a reliable biomarker.

Several possibilities are currently under review to optimize the use of the technology and to solve the potential problems that can be foreseen when transferring a concept from one technology platform to another test method. In the past, a key improvement for the DNA microarrays was the two-color procedure in which cDNA populations from case and control samples were labeled with different colored fluorescent tags and mixed prior to hybridization to the array, allowing the determination of a ratio of the two colors at each feature on the array to be measured [26]. The two-color procedure is by far superior to the measurement of a single value of a captured molecule, since the inherent variability in the spotting process results in some spots having more probe than others or irregular spots. The two-color procedure is not yet integrated for the serum antibody analysis.

Much higher affinity ligands for the disease-related antibodies can be generated using different types of molecules in the discovery phase. In particular, one could evaluate the use of libraries with oligomeric molecules that have more conformational constraints than the very floppy peptoids [28-32]. This modification would eliminate the need for a high level of avidity in order to efficiently retain the AD antibodies from the serum. Another improvement possibility could be to spend more time in the identification of improved derivatives of the primary screening hits through medicinal chemistry-type approaches. Finally, variants of the microarray could be developed that would support avidity-driven binding, be more convenient, and be able to support large-scale studies. The peptoids could be coated onto multimeric surfaces to re-constitute avidity-driven binding even on low-density surfaces.

## Conclusion

Preliminary discovery studies have suggested that there exist IgG antibodies of potential utility as biomarkers of AD. Whether these markers will reach the level of clinical utility will be dependent on much larger validation trials. Moreover, even if these trials are successful, considerable work may remain to render the existing complex assay formats suitable for a clinical test that must accommodate potentially millions of samples. One hopes that the most useful probes discovered through array-based methods could be translated into a more convenient analytical platform. Undoubtedly, over the next few years, we will see intense efforts to evaluate and further document the clinical utility of the IgG biomarkers.

## Abbreviations

AD: Alzheimer’s disease; Aβ: Amyloid-beta; CSF: Cerebrospinal fluid; ELISA: Enzyme-linked immunosorbent assay; IAM: Inhibitor of amyloid; Ig: Immunoglobulin.

## Competing interests

HV is a founder of Biomarkable bvba (Gent, Belgium) and a co-founder of ADx Neurosciences (Gent, Belgium). TK has stock options in Opko Health Inc.
